# Beyond the Fascial Plane: An Open Invitation to Explore Mechanistic Uncertainties and Local Anesthetic Safety

**DOI:** 10.4274/TJAR.2026.262991

**Published:** 2026-10-30

**Authors:** Serkan Tulgar, Alper Kılıçaslan, Özlem Selvi Can, Zekeriyya Alanoğlu

**Affiliations:** 1 Bahçeşehir University Faculty of Medicine, Göztepe Medical Park Hospital, Department of Anaesthesiology and Reanimation, İstanbul, Türkiye; 2 Necmettin Erbakan University Faculty of Medicine, Department of Anaesthesiology and Reanimation, Konya, Türkiye; 3 Ankara University Faculty of Medicine, Department of Anaesthesiology and Reanimation, Ankara, Türkiye; 4 Washington University in St. Louis, Faculty of Medicine, Department of Anaesthesiology, St. Louis, United States

In this issue of the Turkish Journal of Anaesthesiology and Reanimation, we draw particular attention to an increasingly important aspect of regional anaesthesia: the pharmacokinetics and safety of local anesthetics used in fascial plane blocks. While Zengin et al.^1^ provide a comprehensive review of local anesthetic pharmacokinetics in fascial plane blocks, Magoon and Choudhary^2^ raise concerns regarding the safety of local anesthetic dosing in a previously published case report, prompting a response from Çiftçi et al.^3^ As the Editorial Board, we consider these contributions an opportunity to revisit an issue that deserves greater attention.

Over the past decade, the rapid expansion of fascial plane blocks has substantially changed our approach to local anesthetic administration. Many fascial plane blocks use relatively large injectate volumes to facilitate spread toward the intended neural targets. This makes the relationship between volume, concentration, total dose, and systemic absorption particularly relevant to both efficacy and safety. This evolution raises an important question: have our understanding and consideration of local anesthetic pharmacokinetics kept pace with the growing use of high-volume fascial plane blocks? The recent systematic review by De Cassai et al.^4^ examining local anesthetic pharmacokinetics after single-injection erector spinae plane block provides a focused contribution to this discussion.

A growing body of literature indicates that fascial plane blocks can reduce analgesic requirements in selected surgical settings, although their efficacy and sensory effects vary across techniques. Nevertheless, questions regarding the mechanisms underlying these beneficial effects have increasingly attracted the attention of clinicians and researchers. A relatively small but influential group of respected academics has raised the possibility that some of the analgesic effects attributed to fascial plane blocks may, in fact, result from the systemic analgesic action of absorbed local anesthetics rather than solely from their effects on targeted neural structures. Although this hypothesis has not gained widespread acceptance, it has introduced an important dimension to the ongoing scientific discussion. Local and systemic mechanisms need not be mutually exclusive, and their relative contributions may differ across techniques and local anesthetic regimens. Demonstrating systemic absorption alone, however, does not establish that it accounts for the observed analgesic benefit.

Our intention is not to take sides in this debate. We will continue to evaluate studies investigating the clinical efficacy of fascial plane blocks and publish those that meet our scientific and methodological standards. At the same time, we particularly welcome research exploring the pharmacokinetics of local anesthetics and the potential contribution of systemic absorption to the analgesic effects of fascial plane blocks. Such investigations are essential to advancing our understanding of these increasingly popular techniques.

The pursuit of innovation should never come at the expense of patient safety. As fascial plane blocks continue to evolve, we must maintain an appropriate balance between local anesthetic volume, concentration, and total dose. High volume does not necessarily imply a high total dose; these variables should be considered together. Local anesthetic administration should be individualized according to patient characteristics, surgical requirements, and the anatomical properties of the targeted fascial plane.

With patient safety as our foremost priority, we extend an open invitation to researchers to investigate whether, and to what extent, systemic absorption of local anesthetics contributes to the analgesic effects of fascial plane blocks. We particularly welcome experimental and clinical studies that use appropriate comparator groups to help distinguish local from systemic contributions to analgesia, supported by serial plasma concentration measurements, standardized sensory assessment, and clinically relevant analgesic outcomes. We also encourage investigations into the pharmacokinetic profiles of local anesthetics across different fascial plane blocks. As clinicians and researchers, we share this curiosity and look forward to studies that clarify mechanisms, inform dosing, and strengthen patient safety.
